# Advanced Isoconversional Kinetic Analysis for the Elucidation of Complex Reaction Mechanisms: A New Method for the Identification of Rate-Limiting Steps

**DOI:** 10.3390/molecules24091683

**Published:** 2019-04-30

**Authors:** Nicolas Sbirrazzuoli

**Affiliations:** University Côte d’Azur, Institute of Chemistry of Nice, UMR CNRS 7272, 06100 Nice, France; Nicolas.SBIRRAZZUOLI@unice.fr or Nicolas.SBIRRAZZUOLI@univ-cotedazur.fr

**Keywords:** kinetic analysis, isoconversional methods, polymerization mechanisms, curing, epoxy, DSC, thermoanalytical techniques

## Abstract

Two complex cure mechanisms were simulated. Isoconversional kinetic analysis was applied to the resulting data. The study highlighted correlations between the reaction rate, activation energy dependency, rate constants for the chemically controlled part of the reaction and the diffusion-controlled part, activation energy and pre-exponential factors of the individual steps and change in rate-limiting steps. It was shown how some parameters computed using Friedman’s method can help to identify change in the rate-limiting steps of the overall polymerization mechanism as measured by thermoanalytical techniques. It was concluded that the assumption of the validity of a single-step equation when restricted to a given *α* value holds for complex reactions. The method is not limited to chemical reactions, but can be applied to any complex chemical or physical transformation.

## 1. Introduction

The control of polymerization reactions of thermosetting material is of major importance to reach the optimal properties of the cured polymer or composite. These complex reactions often involve several chemical and diffusion steps that make the elucidation of the reaction mechanism very difficult. The use of classical analytical techniques is often limited by high change in the viscosity of the reaction medium during polymerization. Thus, Differential Scanning Calorimetry (DSC) and rheometry have been widely used to monitor complex cure kinetics that depend on both time, temperature and heating (cooling) rate. The final degree of crosslinking, which is correlated to the extent of conversion (*α*), is linked to the temperature program used for curing. In addition, there is a direct link between the final properties of the polymer and (i) the molecular weight, (ii) the extent of conversion as evaluated by DSC and (iii) the glass transition temperature (*T*_g_). As an illustration of this, the Di Benedetto equation shows the link between the glass transition temperature and the extent of conversion [1,2,3]. 

In order to gain more insight into the elucidation of the reaction mechanism of complex reactions, the objective of the work is to show how isoconversional kinetic analysis can provide information on the rate-limiting steps involved in complex chemical reactions or physical transformations by an analysis of the activation energy dependency, called the *E_α_* dependency. In the case of single-step processes, the activation energy computed with an isoconversional method leads to a constant value with the extent of conversion (*α*). Nevertheless, this case is not frequently observed. In the case of complex reactions or complex physical transformations, analysis of the *E*_α_ dependency and its variations indicate the presence of a complex mechanism and may give important insight into change in the rate-limiting steps. For this purpose, a complex chemical polymerization was selected and simulated using data obtained in a previous experimental work [4]. This complex cure is of peculiar interest as it involves several steps consisting of an autocatalytic step, a first-order reaction and a diffusion-controlled part at the end of the reaction. This mechanism was frequently observed in the crosslinking of various epoxy–amine systems and was selected because it represents a good example of reaction complexity [5]. The first stages of the reaction are often controlled by an autocatalytic process, followed by epoxy–amine addition and a diffusion-controlled part after gelation when the viscosity of the reaction medium reaches high values as a result of the high increase of the molecular weight due to crosslinking. The specific polymerization system used as an example was made of 1,3-phenylenediamine (m-PDA) and diglycidyl ether of bisphenol A (DGEBA), obtained from Sigma-Aldrich and used as received. DGEBA has a molecular weight of about 355 g mol^−1^, a glass transition temperature *T*_g_ of about −20 °C (midpoint DSC) and an epoxy equivalent (determined by 1 H NMR) of about 175 g equiv^−1^. The example proposed here to illustrate the method corresponds to a polymerization reaction which is well described by an autocatalytic model for the chemically controlled part of the reaction and by a diffusion model for the end of the reaction. Nevertheless, this procedure can be extended to other complex processes, such as parallel, competitive (consecutive) or other mechanisms.

## 2. Theoretical Part

Isoconversional methods are amongst the more reliable kinetic methods for the treatment of thermoanalytical data, see for example [5,6,7]. The Kinetics Committee of the International Confederation for Thermal Analysis and Calorimetry (ICTAC) has recommended the use of multiple temperature programs for the evaluation of reliable kinetic parameters [6]. The main advantages of isoconversional methods are that they afford an evaluation of the effective activation energy, *E*_α_, without assuming any particular form of the reaction model, *f*(*α*) or *g*(*α*), and that a change in the *E*_α_ variation, called the *E*_α_ dependency, can generally be associated with a change in the reaction mechanism or in the rate-limiting step of the overall reaction rate, as measured with thermoanalytical techniques.

Polymerizations are frequently accompanied by a significant amount of heat released; thus, cure kinetics can be easily monitored by DSC. It is generally assumed that the heat flow measured by calorimetry is proportional to the process rate [5,6,7]. Thus, the extent of conversion at time *t*, *α*_t_, is computed according to Equation (1), as follows:

(1) $$\alpha_{t}=\frac{\int_{t_{i}}^{t}(dQ/dt)\;dt}{\int_{t_{i}}^{t_{f}}(dQ/dt)\;dt}=\frac{Q_{t}}{Q_{tot}}$$

where *t*_i_ represents the first integration bound of the DSC signal and *t*_f_ is the last integration bound selected when the reaction is finished. (d*Q*/d*t*) is the heat flow measured by DSC at time *t*, *Q*_tot_ is the total heat released (or absorbed) by the reaction and *Q*_t_ is the current heat change.

The general form of the basic rate equation is usually written as [5]:

(2) $$\frac{d\alpha }{dt}=A\exp\left(-\frac{E}{RT}\right)f(\alpha )$$

where *T* is the temperature, *f*(α) is the differential form of the mathematical function that describes the reaction model that represents the reaction mechanism, *E* is the activation energy and *A* is the pre-exponential factor. 

The advanced non-linear isoconversional method (NLN) [8,9,10,11] used in this study is presented in Equations (3) and (4) and was derived from Equation (2):

(3) $$\Phi (E_{\alpha })=\sum_{i=1}^{n}\sum_{j\neq i}^{n}\frac{J\left[E_{\alpha },T_{i}(t_{\alpha })\right]}{J\left[E_{\alpha },T_{j}(t_{\alpha })\right]}$$

(4) $$J\left[E_{\alpha },T(t_{\alpha })\right]=\int_{t_{\alpha -\Delta \alpha }}^{t_{\alpha }}\exp\left[\frac{-E_{\alpha }}{RT(t)}\right]\;dt$$

where *E_α_* is the effective activation energy. The *E*_α_ value is determined as the value that minimizes the function Φ(*E*_α_). This non-linear kinetic method (referred as NLN) allows one to handle a set of *n* experiments carried out under different arbitrary temperature programs *T*_i_(*t*) and uses a numerical integration of the integral with respect to the time. For each *i*-th temperature program, the time *t_α,_*_i_ and temperature *T_α,_*_i_ related to selected values of *α* are determined by an accurate interpolation using a Lagrangian algorithm [11,12]. Numerical integration is performed using trapezoidal rule. Several possibilities are proposed for the initial estimate *E*_0_ of *E_α_* in the non-linear procedure. The method developed by Sbirrazzuoli and implemented in his internally generated software can treat any kind of isothermal or non-isothermal data from DSC, calorimetry (C80, for example), Thermogravimetric Analysis (TGA), Dynamic Mechanical Analysis (DMA), or rheometry [9,11,12,13,14,15,16]. This software was used in this study to compute a value of *E_α_* for each value of *α* between 0.02 and 0.98 with a step of 0.02. This advanced non-linear isoconversional method (NLN) was applied in this study.

Another isoconversional method can be derived by the linearization of Equation (2) and is known as Friedman’s method [12,17]:

(5) $$\ln{\left(\frac{d\alpha }{dt}\right)}_{\alpha ,i}=\ln\left[A_{\alpha }\;f(\alpha )\right]-\frac{E_{\alpha }}{RT_{\alpha ,i}}$$

Application of this method requires the knowledge of the reaction rate (d*α*/d*t*)*_α,i_* and of the temperature *T_α,i_* corresponding to a given extent of conversion, for the *i* temperature programs used. The advantages of differential methods such as Friedman’s method (referred as FR) are that they use no approximations and can be applied to any temperature program. As for NLN, the interpolation is made using a Lagrangian algorithm. This does not hold for usual integral methods, but is also the case for the non-linear advanced isoconversional method previously described. Nevertheless, simulations have shown that differential isoconversional methods can sometimes reveal numerical instability [12]; therefore, before using Friedman’s method it was checked that the obtained results were consistent with those obtained with the NLN method.

Equation (5) shows that the intercept of the Friedman’s plot led to the determination of the term [*A*_α_ *f*(*α*)]. This term represents the product between the pre-exponential factor *A*_α_ and the mathematical function *f*(*α*) that describes the reaction mechanism. Once *E_α_* and [*A*_α_ *f*(*α*)] have been evaluated it is possible to compute the reaction rate (d*α*/d*t*) for each value of *α* using Equation (6):

(6) $${\left(\frac{d\alpha }{dt}\right)}_{\alpha }=\left[A_{\alpha }\;f(\alpha )\right]\;\exp\left(-\frac{E_{\alpha }}{RT_{\alpha }}\right)$$

The terms (d*α*/d*t*)_α_, [*A*_α_ *f*(*α*)] and exp(−*E*_α_/*RT*) of Equation (6) were evaluated for each *α*. If the reaction rate increased and the term exp(−*E*_α_/*RT*) decreased (i.e., *E_α_* increased), then it was concluded that an increase of the term [*A*_α_ *f*(*α*)] compensated for the decrease of the exponential term. This corresponds to a change in the pre-exponential factor and/or to the reaction mechanism. Thus, the aim of this work is to show how the analysis of these variations in association with the *E_α_* dependency can be used to identify changes in the reaction mechanism. 

**Figure 1:**
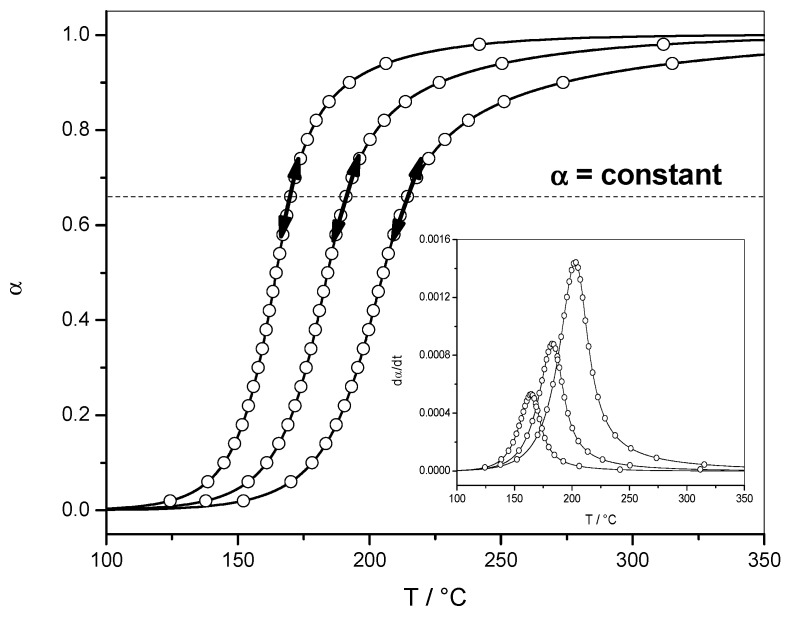
Non-isothermal data. Example of variation of the extent of conversion (*α*) with temperature (*T*). Inset: corresponding variation of the reaction rate (d*α*/d*t*) with temperature. Isoconversional methods are based on the assumption of the hypothesis of a single-step process only for each *α* value and the Arrhenius equation applies to a narrow temperature region related to this *α* value.

Figure 1 show the variation of the extent of conversion (*α*) with temperature (*T*) for three heating rates and the corresponding reaction rate (d*α*/d*t*). Equation (2) is the equation of a single-step process that does not apply to complex polymerization, which is a multi-step process. When applying an isoconversional method, the computations are performed for a constant value of *α*. Thus, Equation (2) is transformed into Equation (6). In this case, the hypothesis of a single-step process is only applied for each constant *α* value used for the computation, which corresponds, for non-isothermal data, to a very narrow temperature range. Therefore, it can be assumed that the validity of a single-step equation for a given *α* value generally holds, even for complex reactions. In addition, the Arrhenius equation is only applied to a narrow temperature region related to this *α* value.

Isoconversional methods require the performance of a series of experiments at different temperature programs and yield the values of effective activation energy *E_α_* as a function of the extent of conversion *α*. A significant variation of *E_α_* with *α* indicates that the process is kinetically complex and the *E*_α_ dependencies evaluated by an isoconversional method allow for meaningful mechanistic and kinetic analyses and for understanding multi-step processes, as well as for reliable kinetic predictions. Model-free isoconversional kinetic methods are a powerful tool to gain information on the reaction complexity through the *E*_α_ dependency determination. A change in the slope of the *E*_α_ dependency may generally be associated with a change in the rate-limiting step of the overall reaction mechanism. Isoconversional methods are based on the isoconversional principle that states that the reaction rate is only a function of temperature for a given constant value of the extent of conversion. Thus, the *E*_α_ dependency can be evaluated without any assumption of the reaction mechanism, as illustrated by Equation (7):

(7) $${\left[\frac{d\ln\left(d\alpha /dt\right)}{dT^{-1}}\right]}_{\alpha }={\left[\frac{d\ln k(T)}{dT^{-1}}\right]}_{\alpha }+{\left[\frac{d\ln f(\alpha )}{dT^{-1}}\right]}_{\alpha }=-\frac{E_{\alpha }}{R}$$

In this equation, the derivative of the term containing *f*(*α*) is zero because each computation is performed for a constant value of *α* (isoconversional methods).

## 3. Data Simulation

Two sets of simulated data were generated and analyzed for non-isothermal conditions using the heating rates of 1, 2 and 4 Kmin^−1^ and for isothermal conditions at four temperatures of 120, 140, 160 and 180 °C. Usually, it is recommended to use three to five temperature programs [6]. In this work only three heating rates were used for the *E*_α_ dependency computations because using three or four heating rates led to the same values as the computation was performed using simulated data.

In the first set (set 1), a complex reaction involving an autocatalytic step, a first-order step and a diffusion process was simulated according to the following equations [18]:

(8) $$k_{D}\left(T,\alpha \right)=D_{0}\exp\left(-\frac{E_{D}}{RT}+K\alpha \right)$$

(9) $$k_{C}\left(T,\alpha \right)=A_{1}\exp\left(-\frac{E_{1}}{RT}\right)+A_{2}\exp\left(-\frac{E_{2}}{RT}\right)\alpha^{m}\;[19,20]$$

(10) $$k_{ef}=k_{C}^{-1}+k_{D}^{-1}\;[18]$$

(11) $$\frac{d\alpha }{dt}=k_{ef}{\left(1-\alpha \right)}^{n}$$

(12) $$\alpha_{i+1}=\alpha_{i}+\int_{t_{i}}^{t_{i+1}}{\left(\frac{d\alpha }{dt}\right)}_{i}dt$$

and using the following parameters [4]: *A*_1_ = 20739.00 s^−1^, *E*_1_ = 70.0 kJ·mol^−1^, *m* = 1, *A*_2_ = 499.00 s^−1^, *E*_2_ = 45.0 kJ·mol^−1^, *n* = 1, *D*_0_ = 1.43 s^−1^, *E*_D_ = 4.4 kJ·mol^−1^, *K* = −7.06. Here *k*_D_, *k*_C_ and *k*_ef_ respectively represent the specific rate constant for diffusion, the rate constant for the chemically controlled reaction and the effective rate constant. *D*_0_ represents the pre-exponential factor of the diffusion-controlled reaction, *A*_1_ the pre-exponential factor for the non-catalyzed reaction and *A*_2_ the pre-exponential factor for the catalyzed reaction. *K* is a constant accounting for the effect of the chemical reaction on the change in diffusivity. *E*_D_, *E*_1_ and *E*_2_ respectively represent the activation energy of the diffusion-controlled reaction, the activation energy of the non-catalyzed reaction and the activation energy of the catalyzed reaction. *m* and *n* are kinetic exponents [19,20].

A second set of data (set 2) involving a first-order step and a diffusion process was simulated according to the same procedure, wherein Equation (9) was replaced by Equation (13):

(13) $$k_{C}(T)=A_{1}\exp\left(\frac{-E_{1}}{RT}\right)$$

and using the following parameters: *A*_1_ = 1762.24 s^−1^, *E*_1_ = 50.0 kJ·mol^−1^, *n* = 1, *D*_0_ = 1.43 s^−1^, *E*_D_ = 4.4 kJ·mol^−1^, *K* = −7.06.

## 4. Results

### 4.1. Autocatalytic Reaction with Diffusion-Controlled Part (Data Set 1)

#### 4.1.1. Reaction Rate and Extent of Conversion for Non-Isothermal and Isothermal Conditions

**Figure 2:**
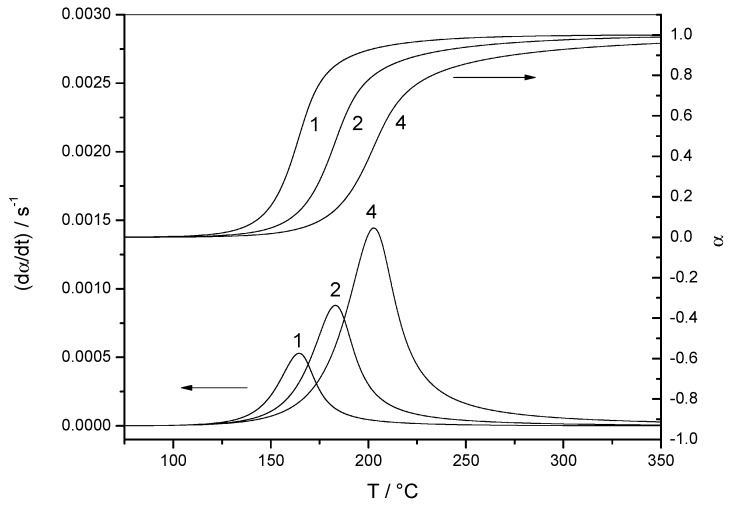
Non-isothermal data. Variation of the reaction rate (d*α*/d*t*) and the extent of conversion (*α*) with temperature (*T*) for data set 1. The heating rate of each experiment (in K min^−1^) is indicated by each curve.

**Figure 3:**
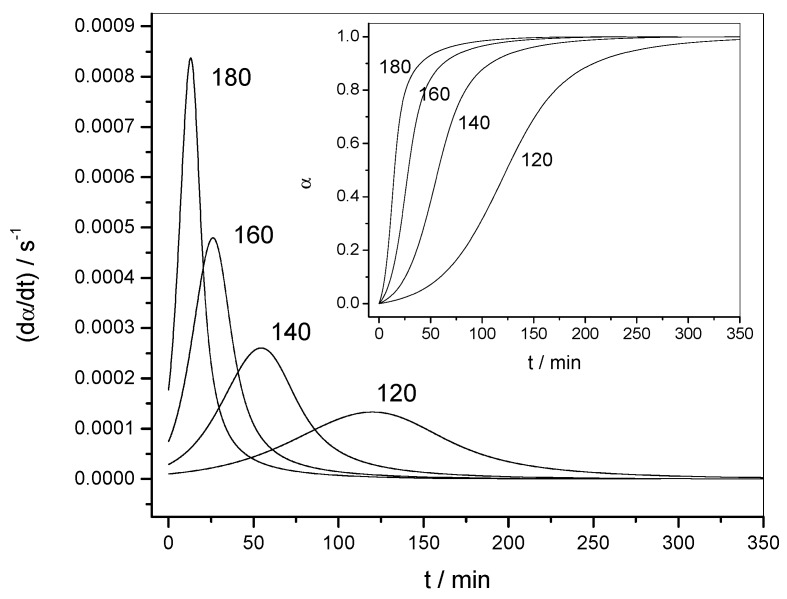
Isothermal data. Variation of the reaction rate (d*α*/d*t*) with time (*t*) for data set 1. Inset: Variation of the extent of conversion (*α*) with time (*t*). The temperature of each experiment (in °C) is indicated by each curve.

Figure 2 shows the variation of the reaction rate and of the extent of conversion with temperature for three heating rates (non-isothermal data). The curves shifted to higher temperatures when the heating rate was increased. Figure 3 shows the variation of the reaction rate and of the extent of conversion with time for four temperatures (isothermal data). It can be observed that the maximum of the reaction rate was not obtained for *α* = 0, as would be the case for a reaction order mechanism. Another characteristic feature of the autocatalytic model is that the isothermal *α*–*t* curves presented typical sigmoidal shapes, as shown in Figure 3.

#### 4.1.2. Dependence of the Effective Activation Energy and of the Pre-Exponential Factor

**Figure 4:**
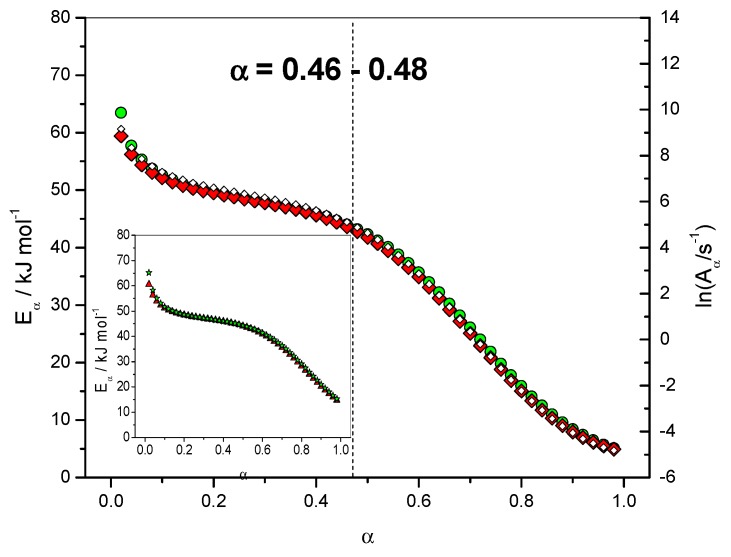
Dependence of the effective activation energy (*E_α_*) and pre-exponential factor (ln*A_α_*) with the extent of conversion (*α*). Non-isothermal data. Open lozenges: pre-exponential factor, red solid lozenges: *E_α_* computed with FR method, green circles: *E_α_* computed with NLN method. Inset: isothermal data. Solid triangles: *E_α_* computed with FR method, solid stars: *E_α_* computed with NLN method.

The dependence of the effective activation energy (*E_α_*) with the extent of conversion (*α*) is presented in Figure 4 (left axis). Note that FR and NLN methods gave similar results in this case. The complexity of the mechanism is perfectly reflected by the important *E*_α_ dependence observed. The first value obtained was *E*_α_ = 63.4 kJ·mol^−1^ for *α* = 0.02 with the NLN method. This value is close to the value of *E*_1_ = 70.0 kJ·mol^−1^ used in the simulation for the activation energy of the uncatalyzed reaction. For *α* = 0.001 this value would be *E*_α_ = 69.0 kJ·mol^−1^ which is very close to *E*_1_. The lowest activation energy value was *E*_α_ = 5.1 kJ·mol^−1^ for *α* = 0.98 (NLN method), which is very close to the activation energy of diffusion (*E*_D_ = 4.4 kJ·mol^−1^). 

**Figure 5:**
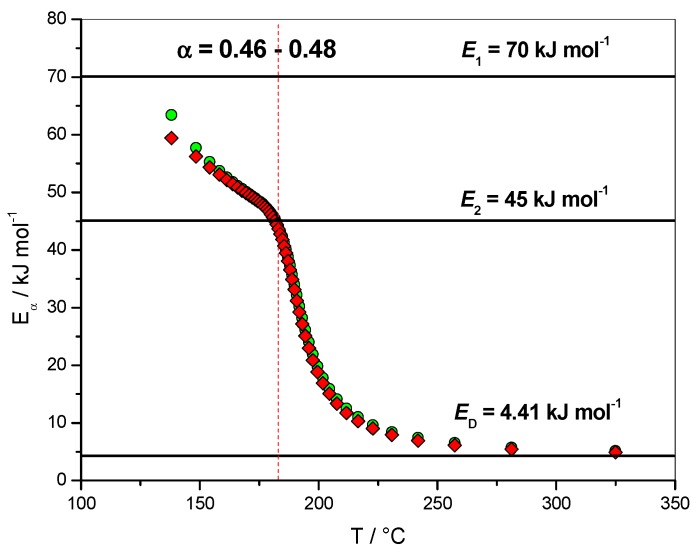
Non-isothermal data. Dependence of the effective activation energy (*E_α_*) with temperature. Red solid lozenges: *E_α_* computed with FR method, green open circles: *E_α_* computed with NLN method.

Figure 4 (right axis) give the results obtained for the dependence of the logarithm of the pre-exponential factor (ln*A*_α_) with the extent of conversion (*α*). This dependence was computed using the compensation parameters method described by Sbirrazzuoli [11]. For this computation the model-fitting method of Achar–Brindley–Sharp was used in the interval 0.10 < *α* < 0.40 to evaluate the relationship between *E* and ln*A*. A good correlation (*r*^2^ = 0.99903) between these two parameters was obtained using the models F1 (Mampel, first order), A2 (Avrami–Erofeev), D3 (three-dimensional diffusion), R3 (contracting sphere) and D2 (two-dimensional diffusion) of ref. [6]. For *α* = 0.02, ln(*A*_α_/s^−1^) was found to be 9.16, which is very close to the value used in the simulation, i.e., ln(*A*_1_/s^−1^) = 9.94. The lowest value for ln(*A*_α_/s^−1^) was −4.82, which can be associated with ln(*D*_0_/s^−1^) = 0.36. The dependence of the effective activation energy (*E_α_*) with temperature (*T*) is presented in Figure 5 in association with the corresponding activation energies of the different steps, i.e., *E*_1_, *E*_2_ and *E*_D_.

#### 4.1.3. Variation of the Reaction Rate with the Extent of Conversion

**Figure 6:**
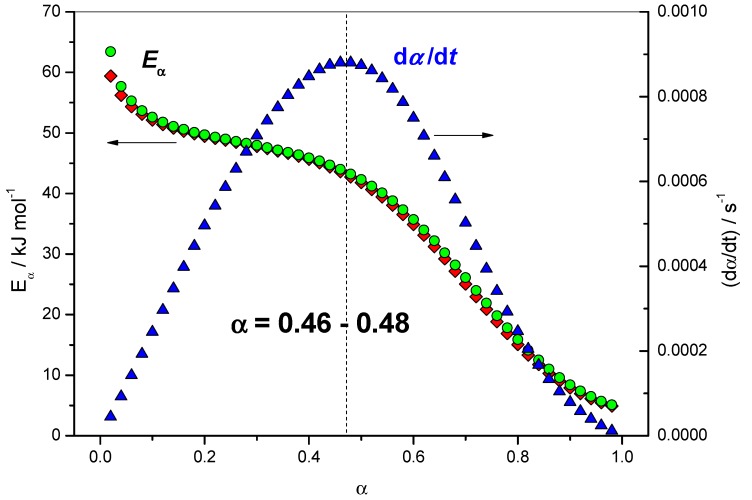
Dependence of the effective activation energy (*E_α_*) with the extent of conversion (*α*). Red lozenges: *E_α_* computed with FR method, green circles: *E_α_* computed with NLN method, blue triangles: (d*α*/d*t*) computed as the product of [*A*_α_ *f*(α)] by exp[−*E*_α_/(*RT*_α_)] (Equation (6)).

The terms [*A*_α_ *f*(α)] and exp[−*E*_α_/(*RT*_α_)] can be evaluated using Equation (6), then a value of the reaction rate can be computed for each extent of conversion as the product of [*A*_α_ *f*(α)] by exp[−*E*_α_/(*RT*_α_)]. Figure 6 shows the relation between the dependence of the effective activation energy (*E_α_*) with the extent of conversion (*α*) and the reaction rate (d*α*/d*t*). The maximum of the reaction rate (d*α*/d*t*) was located in the range of 0.46 < *α* < 0.48. Figure 6 shows that this corresponds to a change in the slope of the *E*_α_ dependence.

**Figure 7:**
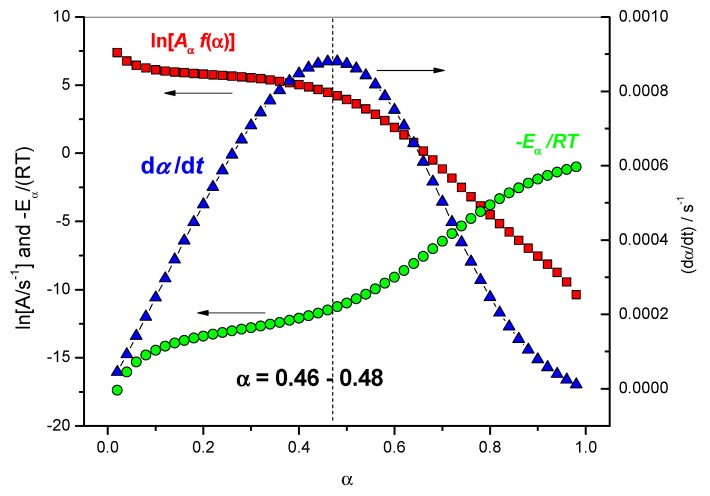
Red squares: variation of ln [*A*_α_ *f*(α)] with *α*, green circles: variation of −*E*_α_/(*RT*_α_) with *α*, blue triangles: variation of (d*α*/d*t*) computed as the product of [*A*_α_ *f*(α)] by exp[−*E*_α_/(*RT*_α_)] with *α*.

The comparison of the terms [*A*_α_ *f*(α)], exp[−*E*_α_/(*RT*_α_)] and (d*α*/d*t*) can be used to identify rate-limiting steps in the overall reaction rate. The principle of this method is explained below and illustrated by Figure 7. From *α* = 0.02 to 0.46, (d*α*/d*t*) increases and the term [*A*_α_ *f*(*α*)] decreases, so the increase of (d*α*/d*t*) is attributed to the increase of the term exp[−*E*_α_/(*RT*_α_)], i.e., a decrease of *E*_α_. Thus, the increase of the rate is mainly due to a favorable energetic term. When *α* ≥ 0.48, (d*α*/d*t*) decreases, the term exp[−*E*_α_/(*RT*_α_)] still increases and the term [*A*_α_ *f*(*α*)] still decreases. This indicates that the term [*A*_α_ *f*(*α*)] dominates and corresponds to a change in the rate-limiting step at this stage of the reaction. The decrease of the rate is attributed to a change in the mechanism, which may originate from a change in *f*(*α*) or from an entropic change (*A*_α_). This entropic change may be due to a change in configuration or a decrease in the efficiency of collisions. Thus, it is identified as a change in the rate-limiting step in the overall reaction rate for *α* = 0.46–0.48. Table 1 gives some values of the various terms of Figure 7 used to identify a change in the rate-limiting step for 0.46 < *α* < 0.48.

Once the change in the rate-limiting step was identified for *α* = 0.46, an analysis of Figure 4 showed that ln(*A*_α_/s^−1^) = 5.11 and *E_α_* = 44.0 kJ·mol^−1^ for *α* = 0.46, which are close to the values used in the simulation for the catalyzed reaction, i.e., ln(*A*_2_/s^−1^) = 6.21 and *E*_2_ = 45.0 kJ·mol^−1^. The change in the curvature of the *E*_α_ dependence occurred exactly in this region of *α* = 0.46–0.48, as seen in Figure 4, Figure 5 and Figure 6. This result shows that, in addition to giving information on the rate-limiting steps, the *E*_α_ and ln(*A*_α_) dependencies can be used as estimate values of kinetic parameters to be used in a non-linear fitting procedure [9].

#### 4.1.4. Variation of the Rate Coefficients with Extent of Conversion

**Figure 8:**
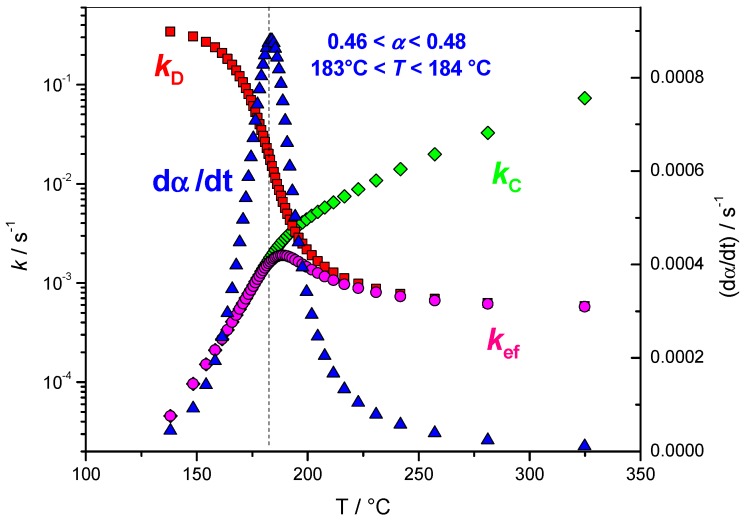
Blue triangles: variation of (d*α*/d*t*) computed as the product of [*A*_α_ *f*(α)] by exp[−*E*_α_/(*RT*_α_)] with temperature, red squares: variation of *k*_D_ with temperature, green lozenges: variation of *k*_C_ with temperature, magenta circles: variation of *k*_ef_ with temperature.

Figure 8 shows the variation of the rate coefficients *k*_D_, *k*_C_ and *k*_ef_ with the extent of conversion (*α*) *k*_D_ always decreased with *α*, while *k*_C_ always increased. Initial values of *k*_D_ were high, while they were low for *k*_C_. This is the opposite at the end where the values of *k*_D_ were lower than the values of *k*_C_. This is in perfect agreement with a chemical control at the beginning of the reaction (*k*_C_ << *k*_D_) followed by a diffusion control at the end (*k*_D_ << *k*_C_). The variation of *k*_ef_ is more complex, as reflected by Equation (10), showing an increasing trend at the beginning and a decreasing tendency at the end. The previously reported value of *α* = 0.46–0.48 (*T*~183 °C), attributed to a change in the rate-limiting step, corresponded to the point at which *k*_C_ started to become higher than *k*_ef_ and *k*_D_/*k*_C_ < 10 (9.6). Generally, it is estimated that a factor of 100 is the minimum required to neglect one reaction to another. This value was obtained for *α* = 0.24 (*T*~173 °C), *k*_D_/*k*_C_ ≈ 100 (102.7).

#### 4.1.5. Variation of the Overall Rate Coefficient *k*(*T*) and of the Effective Rate Coefficient *k_ef_*(*T*) with Reciprocal Temperature

**Figure 9:**
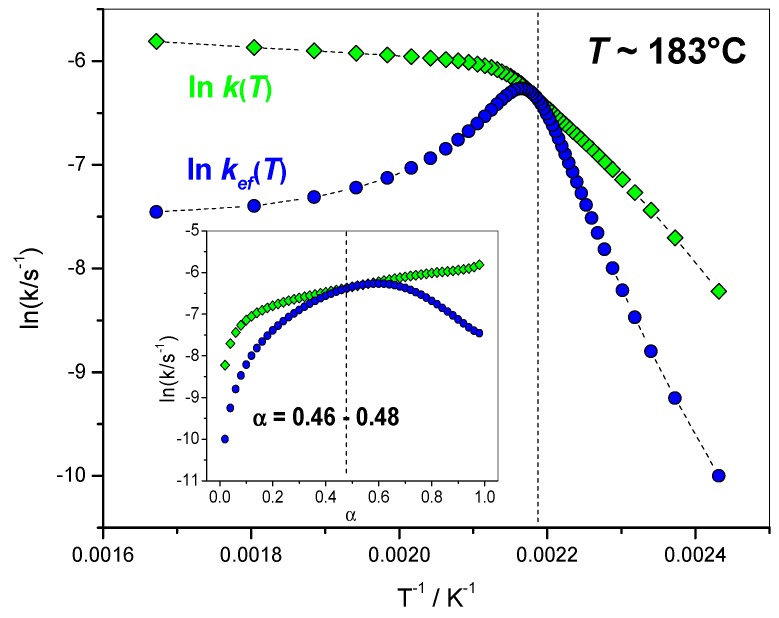
Green lozenges: variation of ln *k*(*T*) computed as ln *k*(*T*) = ln(*A*_α_)–*E*_α_/(*RT*_α_) as a function of reciprocal temperature, blue circles: variation of ln *k_ef_* (*T*) computed using Equation (10) as a function of reciprocal temperature.

The variations of ln *k*(*T*) and ln *k_ef_* (*T*) with reciprocal temperature are presented in Figure 9. The values of *α* = 0.46–0.48 corresponded to the temperature at which the two rate constants were very close. The closest values were reached for *α* = 0.54.

#### 4.1.6. Fit of the E_α_ Dependence with the Sourour and Kamal and Diffusion Models

The isoconversional principle (Equation (7)) was applied to the autocatalytic equation of Sourour and Kamal (Equation (9)). This model is used to describe the initial stages of the reaction when it is chemically controlled [4,5,18]:

(14) $$E_{\alpha }=\frac{k_{1}\left(T\right)E_{1}+k_{2}\left(T\right)E_{2}\;\alpha^{m}}{k_{1}\left(T\right)+k_{2}\left(T\right)\;\alpha^{m}}$$

(15) $$E_{\alpha }=\frac{\left(A_{1}/A_{2}\right)\exp\left(-E_{1}/RT\right)E_{1}+\exp\left(-E_{2}/RT\right)E_{2}\;\alpha^{m}}{\left(A_{1}/A_{2}\right)\exp\left(-E_{1}/RT\right)+\exp\left(-E_{2}/RT\right)\;\alpha^{m}}$$

The isoconversional principle (Equation (7)) was also applied to the diffusion model (Equation (8)) used to describe the end of the reaction [4,5,18]:

(16) $$E_{\alpha }=\frac{k\left(T\right)E_{D}+k_{D}\left(T,\alpha \right)E_{2}}{k\left(T\right)+k_{D}\left(T,\alpha \right)}$$

(17) $$E_{\alpha }=\frac{\left(A_{2}/D_{0}\right)\exp\left(-E_{2}/RT\right)E_{D}+\exp\left(-E_{D}/RT+K\alpha \right)E_{2}}{\left(A_{2}/D_{0}\right)\exp\left(-E_{2}/RT\right)+\exp\left(-E_{D}/RT+K\alpha \right)}$$

Note that, according to Equation (15), at the lowest extent of conversion (*α* → 0) *E*_α_ tends toward the activation energy of the uncatalyzed reaction (*E*_α_ → *E*_1_), in agreement to what was reported in the analysis of Figure 4. 

**Figure 10:**
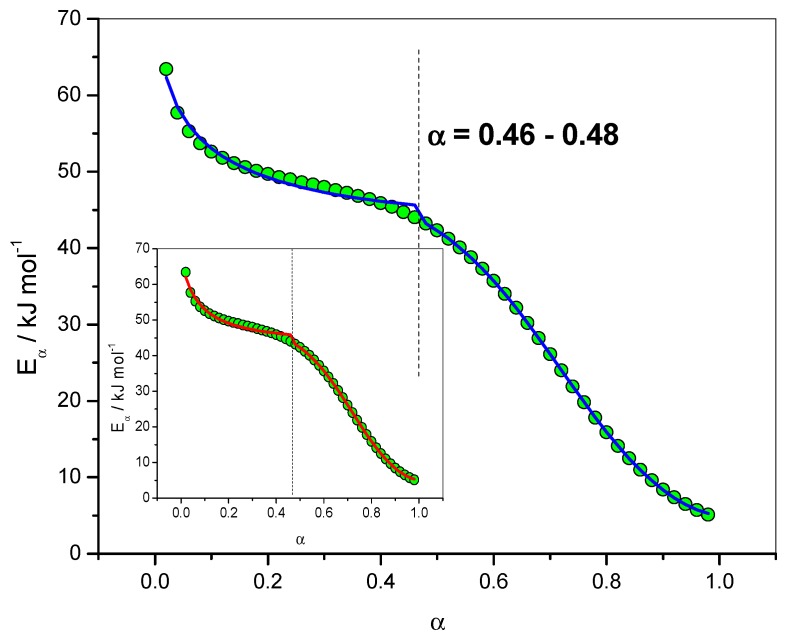
Circles: *E*_α_ dependency obtained with NLN method, line: fit using the autocatalytic model for 0.02 < *α* < 0.46 and the diffusion model for 0.48 < *α* < 0.98 with the parameters of Table 2. Inset: same plot for the data of Table 3.

The results of the fit of Equations (15) and (17) are given in Table 2. Some differences were observed between the simulated values and the values resulting from the non-linear fit. A higher discrepancy was obtained for *A*_1_/*A*_2_ (5918.03). This value should be 41.56. Nevertheless, a good agreement was found between the simulated and experimental data resulting from the non-linear fit for the other parameters. The result of this fit is presented in Figure 10. 

Equations (15) and (17) can be expanded to allow computations of the pre-exponential factors, as proposed by Sbirrazzuoli in [4]. The resulting Equations (18) and (19) can be fitted to estimate *A*_1_, *A*_2_ and *D*_0_. The results are given in Table 3.

(18) $$E_{\alpha }=\frac{A_{1}\exp\left(-E_{1}/RT\right)E_{1}+A_{2}\exp\left(-E_{2}/RT\right)E_{2}\alpha^{m}}{A_{1}\exp\left(-E_{1}/RT\right)+A_{2}\exp\left(-E_{2}/RT\right)\alpha^{m}}$$

(19) $$E_{\alpha }=\frac{A_{2}\exp\left(-E_{2}/RT\right)E_{D}+D_{0}\exp\left(-E_{D}/RT+K\alpha \right)E_{2}}{A_{2}\exp\left(-E_{2}/RT\right)+D_{0}\exp\left(-E_{D}/RT+K\alpha \right)}$$

It can be seen that the parameters are in very good agreement with the reference values and the values of *A*_1_, *A*_2_ are very close the reference values in this case. For the initial part of the reaction, the restriction of the fit to the interval 0.02 < *α* < 0.24 resulted in an improved accuracy (lower *MSSD*). This confirms the previous statement that when *k*_D_/*k*_C_ ≈ 100 it is possible to neglect the diffusion reaction, while for higher temperatures (or values of *α*) it is not completely negligible. Although the fit was better for parameters of Table 3 in comparison with those of Table 2, it is impossible to see the difference on the graph (inset of Figure 10). For the autocatalytic model of Sourour and Kamal, the accuracy of the fit was improved by addition of more flexibility when moving from Equation (15) to Equation (18). Nevertheless, this increased the possibilities of reaching local minima, resulting in several sets of parameters leading to an accurate fit of the data. The use of parameters estimated by the advanced isoconversional method, as initial values of the non-linear fit, greatly facilitated the achievement of meaningful parameters and not only fitting parameters, especially for the initial part of the reaction. However, the existence of local minima is a problem that cannot be underestimated and that is difficult to avoid when fitting complex mechanisms which involve many kinetic parameters to be determined using non-linear fits. This is less problematic for the end of the reaction, i.e., for the fit of Equation (8). The use of a genetic algorithm could be an efficient method to avoid this kind of problem [21].

### 4.2. First-Order Reaction with Diffusion-Controlled Part (Data Set 2)

**Figure 11:**
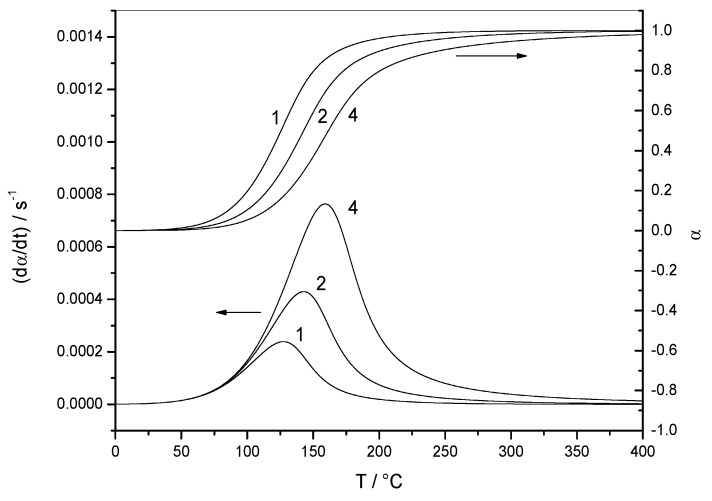
Non-isothermal data. Variation of the reaction rate (d*α*/d*t*) and extent of conversion (*α*) with temperature (*T*) for data set 2. The heating rate of each experiment (in K min^−1^) is indicated by each curve.

**Figure 12:**
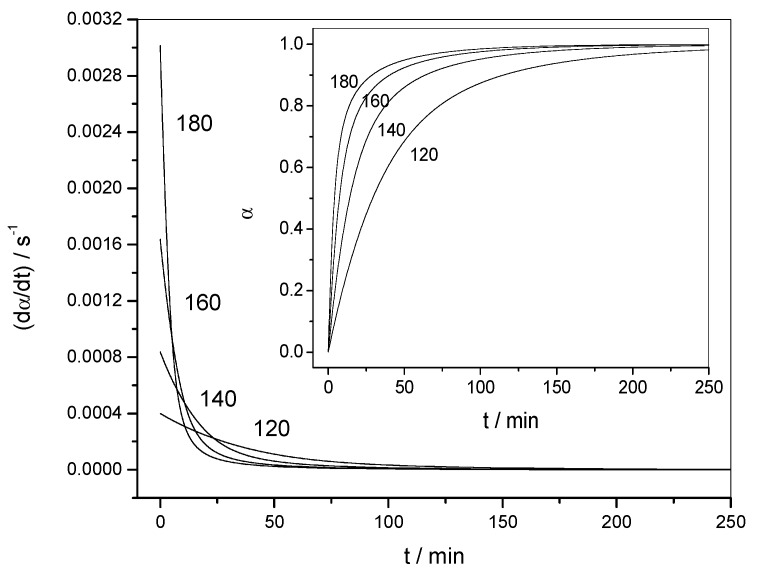
Isothermal data. Variation of the reaction rate (d*α*/d*t*) with time (*t*) for data set 2. Inset: Variation of the extent of conversion (*α*) with time (*t*). The temperature of each experiment (in °C) is indicated by each curve.

Figure 11 shows the variation of the reaction rate and of the extent of conversion with temperature for three heating rates. In comparison with what was observed for data set 1, which include an autocatalytic step (Figure 2), the shift to higher temperatures upon increasing the heating rate was much lower in this case. This shows the difference between the reaction order and the autocatalytic mechanism for non-isothermal data. Figure 12 shows the variation of the reaction rate and of the extent of conversion with time for four temperatures (isothermal data). As expected, the maximum of the reaction rate was obtained for *α* = 0 in this case and the isothermal *α*–*t* curves presented the characteristic shapes of a reaction order model.

**Figure 13:**
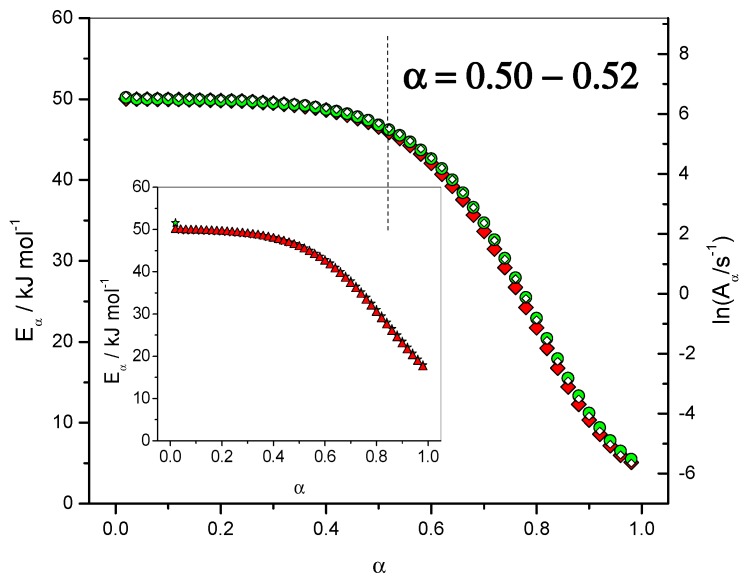
Dependence of the effective activation energy (*E_α_*) and pre-exponential factor (ln*A_α_*) with the extent of conversion (*α*). Non-isothermal data. Open lozenges: pre-exponential factor computed using compensation parameters method, red lozenges: *E_α_* computed with FR method, green circles: *E_α_* computed with NLN method. Inset: isothermal data. Red triangles: *E_α_* computed with FR method, green stars: *E_α_* computed with NLN method.

**Figure 14:**
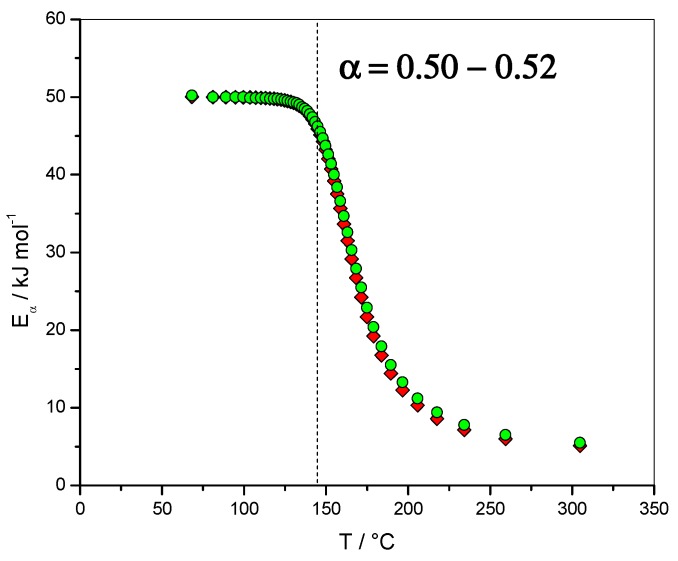
Non-isothermal data. Dependence of the effective activation energy (*E_α_*) with temperature (*T*). Red lozenges: *E_α_* computed with FR method, green circles: *E_α_* computed with NLN method.

The dependence of the effective activation energy (*E_α_*) with extent of conversion (*α*) is presented in Figure 13 (left axis) and the dependence with temperature is presented in Figure 14. Note that FR and NLN methods gave similar results in this case. The complexity of the mechanism is perfectly reflected by the important *E*_α_ dependence observed. The first value obtained was around 50 kJ·mol^−1^, which is in perfect agreement with the value of the activation energy of the reaction order reaction *E*_1_ (50.0 kJ·mol^−1^). Figure 13 (right axis) gives the results obtained for the dependence of the logarithm of the pre-exponential factor (ln*A*_α_/s^−1^) with the extent of conversion (*α*). ln(*A*_α_/s^−1^) was found to be 6.61 and *E_α_* = 50.2 kJ·mol^−1^ for *α* = 0.02, which are close to the values used in the simulation, i.e., ln(*A*_1_/s^−1^) = 7.47 and *E*_1_ = 50.0 kJ·mol^−1^. For *α* = 0.98, ln(*A*_α_/s^−1^) was found to be −5.65 and *E_α_* = 5.5 kJ·mol^−1^, which are also close to the values used in the simulation, i.e., ln(*D*_0_/s^−1^) = 0.36 and *E*_D_ = 4.4 kJ·mol^−1^. 

Application of the compensation parameters method [11] in the interval 0.05 < *α* < 0.25 (Achar-Brindley–Sharp’s differential method, models F1, A3, A2, D3, *r*^2^ = 0.999999) permit the identification of the F1 model (Mampel, first order) and the kinetic parameters of the reaction order reaction. The values found were ln(*A*/s^−1^) = 7.45 and *E* = 49.9 kJ·mol^−1^, while the kinetic parameters used in the simulation were ln(*A*_1_/s^−1^) = 7.47 and *E*_1_ = 50.0 kJ·mol^−1^.

In the case of two reactions with similar activation energies, the identification of the contribution of each individual reaction using the *E*_α_ dependency could be more difficult. Nevertheless, it is highly probable that these reactions would have different pre-exponential factors. In this case, we proposed to identify the complexity of the overall process by analyzing the *A*_α_ dependency computed with the compensation method. 

## 5. Conclusions

A kinetic model has been proposed that simulates complex polymerizations with high accuracy. The first stages of the reaction were described by an autocatalytic process, followed by epoxy–amine addition and a diffusion-controlled model at the end of the reaction. Isoconversional methods—based on the assumption of the hypothesis of a single-step process only for each *α* value and the application of the Arrhenius equation to a very narrow temperature region related to this *α* value—give important insights into the change in the rate-limiting steps by analysis of the *E_α_* dependency and its variations. In addition to this, the comparisons of the terms (d*α*/d*t*), [*A*_α_ *f*(*α*)] and exp[−*E*_α_/(*RT*_α_)] evaluated by Friedman’s method can help to identify the change in the rate-limiting steps of the overall mechanism as measured by thermoanalytical techniques. It was also concluded that the assumption of the validity of a single-step equation, when restricted to a given *α* value, holds for complex reactions. 

## Figures and Tables

**Table 1:** Some values of the various terms used to identify a change in the rate-limiting step.

*T*_α_/°C	*E*_α_ (FR)/kJ·mol^−1^	*α*	ln (*A*_α_/s^−1^)	[*A*_α_ *f*(α)]/s^−1^	exp(−*E*_α_/(*RT*_α_)	(dα/d*t*)/s^−1^
181.56	45.05	0.42	5.47	130.25	6.632 × 10^−6^	8.638 × 10^−4^
182.34	44.39	0.44	5.30	108.30	8.076 × 10^−6^	8.746 × 10^−4^
183.11	43.63	0.46	5.11	87.43	1.007 × 10^−5^	8.802 × 10^−4^
183.89	42.76	0.48	4.89	68.30	1.289 × 10^−5^	8.801 × 10^−4^
184.66	41.78	0.50	4.63	51.45	1.699 × 10^−5^	8.740 × 10^−4^
185.45	40.68	0.52	4.35	37.26	2.313 × 10^−5^	8.618 × 10^−4^

**Table 2:** Parameters obtained by fitting Equations (15) and (17).

2 < *α* < 46%	***A*_1_/*A*_2_**	***E*_1_/kJ·mol^−1^**	***E*_2_/kJ·mol^−1^**	***m***	***MSSD*^a^**
Autocatalytic	5918.03	79.8	38.2	0.97	0.4435
48 < *α* < 98%	***A*/*D*_0_**	***E*_2_/kJ·mol^−1^**	***E*_D_/kJ·mol^−1^**	***K***	***MSSD*^a^**
Diffusion	362.67	48.9	4.9	−7.84	0.0023

^a^ Mean of the sum of squared deviations $MSSD=\left(1/n\right)\sum_{i=1}^{n}{\left(E_{calc}-E_{ref}\right)}^{2}/E_{ref}$.

**Table 3:** Parameters obtained by fitting Equations (18) and (19).

	*A*_1_/s^−1^	*A*_2_/s^−1^	*E*_1_/kJ·mol^−1^	*E*_2_/kJ·mol^−1^	*m*	*MSSD* ^a^
2 < *α* < 46%	20756.17	498.52	67.6	42.6	1.2	0.6011
2 < *α* < 24%	20258.31	510.84	76.1	46.7	1.3	0.0345
	***A*_2_/s^−1^**	***D*_0_/s^−1^**	***E*_2_/kJ·mol^−1^**	***E*_D_/kJ·mol^−1^**	***K***	***MSSD*^a^**
48 < *α* < 98%	498.9	1.43	48.8	4.9	−7.87	0.0024

^a^ Mean of the sum of squared deviations $MSSD=\left(1/n\right)\sum_{i=1}^{n}{\left(E_{calc}-E_{ref}\right)}^{2}/E_{ref}$.

## References

[1] Pascault J.P., Sautereau H., Verdu J., Williams R.J.J. (2002). Thermosetting polymers.

[2] Hale A., Macosko C.W., Bair H.E. (1991). Glass Transition Temperature as a Function of Conversion in Thermosetting Polymers. Macromolecules.

[3] Pascault J.P., Williams R.J.J. (1990). Glass Transition Temperature Versus Conversion Relationships for Thermosetting Polymers. J. Polym. Sci. Part B Polym. Phys..

[4] Alzina C., Sbirrazzuoli N., Mija A. (2010). Hybrid Nanocomposites: Advanced Nonlinear Method for Calculating Key Kinetic Parameters of Complex Cure Kinetics. J. Phys. Chem. B.

[5] Vyazovkin S., Sbirrazzuoli N. (2006). Isoconversional kinetic analysis of thermally stimulated processes in polymers. Macromol. Rapid Comm..

[6] Vyazovkin S., Burnham A.K., Criado J.M., Pérez-Maqueda L.A., Popescu C., Sbirrazzuoli N. (2011). ICTAC kinetics committee recommendations for performing kinetic computations on thermal analysis data. Thermochim. Acta.

[7] Vyazovkin S. (2015). Isoconversional Kinetics of Thermally Stimulated Processes.

[8] Vyazovkin S. (1997). Evaluation of activation energy of thermally stimulated solid-state reactions under arbitrary variation of temperature. J. Comput. Chem..

[9] Sbirrazzuoli N., Vincent L., Vyazovkin S. (2000). Comparison of several computational procedures for evaluating the kinetics of thermally stimulated condensed phase reactions. Chemometr. Intell. Lab.

[10] Vyazovkin S. (2001). Modification of the integral isoconversional method to account for variation in the activation energy. J. Comput. Chem..

[11] Sbirrazzuoli N. (2013). Determination of pre-exponential factors and of the mathematical functions *f*(α) or *G*(α) that describe the reaction mechanism in a model-free way. Thermochim. Acta.

[12] Sbirrazzuoli N. (2007). Is the Friedman method applicable to transformations with temperature dependent reaction heat?. Macromol. Chem. Phys..

[13] Sbirrazzuoli N., Vincent L., Vyazovkin S. (2000). Electronic solution to the problem of a kinetic standard for DSC measurements. Chemometr. Intell. Lab.

[14] Sbirrazzuoli N., Brunel D., Elegant L. (1992). Different kinetic equations analysis. J. Therm. Anal..

[15] Sbirrazzuoli N., Girault Y., Elegant L. (1997). Simulations for evaluation of kinetic methods in differential scanning calorimetry. Part 3—peak maximum evolution methods and isoconversional methods. Thermochim. Acta.

[16] Falco G., Guigo N., Vincent L., Sbirrazzuoli N. (2018). FA polymerization disruption by protic polar solvent. Polymers.

[17] Friedman H.L. (1964). Kinetics of thermal degradation of char-forming plastics from thermogravimetry. Application to a phenolic plastic. J. Polym. Sci. Part C.

[18] Vyazovkin S., Sbirrazzuoli N. (1996). Mechanism and kinetics of epoxy-amine cure studied by differential scanning calorimetry. Macromolecules.

[19] Ryan M.E., Dutta A. (1979). Kinetics of epoxy cure: A rapid technique for kinetic parameter estimation. Polymer.

[20] Sourour S., Kamal M.R. (1976). Differential scanning calorimetry of epoxy cure: Isothermal cure kinetics. Thermochim. Acta.

[21] Yamini G., Shakeri A., Vafayan M., Zohuriaan-Mehr M.J., Kabiri K., Zolghadr M. (2019). Cure kinetics of modified lignosulfonate/epoxy blends. Thermochim. Acta.

